# HBV Treatment in Turkey: The Value of Hepatitis B Surface Antigen Quantification of Chronic Hepatitis B Patients in the Long-term Follow-up—A Single-center Study

**DOI:** 10.5005/jp-journals-10018-1218

**Published:** 2017-05-05

**Authors:** Hasan Ozkan

**Affiliations:** Department of Gastroenterology, Ankara University School of Medicine, Ankara, Turkey

**Keywords:** Chronic hepatitis B, Follow-up, Hepatitis B surface antigen.

## Abstract

Hepatitis B surface antigen (HBsAg) seems to have significant clinical implications to assess the prognosis of chronic hepatitis B (CHB). We assessed HBsAg levels serially in patients with CHB in a single center in Turkey.

**How to cite this article:** Ozkan H. HBV Treatment in Turkey: The Value of Hepatitis B Surface Antigen Quantification of Chronic Hepatitis B Patients in the Long-term Follow-up—A Single-center Study. Euroasian J Hepato-Gastroenterol 2017;7(1):82-83.

## STUDY DETAILS

In Turkey, like in the rest of the world, chronic hepatitis B (CHB) is one of the most important health problems. The hepatitis B virus prevalence is 2.3% in Turkey. This disease progresses into cirrhosis and hepatocellular carcinoma (HCC) among 20 to 40% of CHB patients. There is no cure for CHB, but this dynamic disease can be controlled. To this end, lifelong monitoring is required. The disease must be inactive. Immune control phase and resolved CHB phase are desired. There is sustained immune control in these phases. It is best if these phases are achieved under 40 years of age. In this case, there will be a low risk of cirrhosis. It is best if these phases are achieved under 50 years of age. In this case, there will be a low risk of HCC. Hepatitis B surface antigen (HBsAg) seroclearance is the most reliable marker of immune control associated with an excellent long-term prognosis, if there is lack of cirrhosis at the time of clearance. Also, HCC, decompensation, and death risk will be lower. The HBsAg clearance improves survival rates. Serum HBsAg is the indicator of the number of infected hepatocytes. Serum HBsAg levels are correlated with intrahepatic covalently closed circular deoxyribonucleic acid (DNA) levels. So, these levels show infected cells. The HBsAg quantification (qHBsAg) can predict spontaneous HBsAg loss in HBV carriers. A serum qHBsAg level of less than 100 IU/mL at 1 year post-hepatitis B e antigen (HBeAg) seroconversion can predict HBsAg loss within 6 years. A serum qHBsAg level of less than 10 IU/mL is the strongest predictor of HBsAg loss in HBeAg-negative patients who have an HBV DNA level of less than 2,000 IU/mL. A Taiwanese study shows that when a rapid qHBsAg declines below 1,000 IU/mL within 1 year during treatment, HBsAg seroconversion becomes 24.3% if qHBsAg levels are below 100 IU/mL; HBsAg seroconversion becomes 4.4% if qHBsAg levels are between 100 and 1,000 IU/mL within 6 years.

We have followed 1,274 HBsAg-positive cases. The longest follow-up period is 19.2 years and the average follow-up period is 8 years. Of these cases, 13.5% are HBeAg positive, while 86.5% of them are negative; 5.9% have an anti-delta positivity. Among the negative cases, a decline of qHBsAg is seen in 135 cases (54 women, 81 men), where the average age is 44.8 years. A total of 768 cases have inactive HBsAg carrier and the seroclearance rate is 4%. An HBsAg seroclearance rate of 6.6% is observed in HBeAg-negative patients using antiviral drugs. Furthermore, an HBsAg seroclearance rate of 13.2% is found in patients using interferon (IFN). Approximately 3 years is required for HBV DNA negativity, after qHBsAg declines. Likewise, approximately 6.68 years is required for HBsAg clearance after HBV DNA negativity. Seroclearance of HBsAg was seen in 21% of HBeAg-positive patients with pegylated IFN treatment.

## CONCLUSION

Natural course of CHB is complex with various phases and outcomes. Serum HBsAg loss is closest to clinical cure. Quantitative serum HBsAg levels may predict response to treatments and may help to tailor treatment duration. It is the strongest predictor of HBsAg loss, when the qHBsAg level is less than 10 and 100 IU/mL.

## References

[1] Tapia J, Murguia R, Garcia G, de los Monteros PE, Onate E (1999). Jejunostomy: techniques, indications, and complications. World J Surg.

[2] Young MT, Troung H, Gebhart A, Shih A, Nguyen NT (2016). Outcomes of laparoscopic feeding jejunostomy tube placement in 299 patients. Surg Endosc.

[3] Blumenstein I, Shastri YM, Stein J (2014). Gastroenteric tube feeding: techniques, problems and solutions. World J Gastroenterol.

[4] Braunschweig CL, Levy P, Sheean PM, Wang X (2001). Enteral compared with parenteral nutrition: a meta-analysis. Am J Clin Nutr.

[5] Pritchard C, Duffy S, Edington J, Pang F (2006). Enteral nutrition and oral nutrition supplements: a review of the economics literature. JPEN J Parenter Enteral Nutr.

[6] Kwon RS, Banerjee S, Desilets D, Diehl DL, Farraye FA, Kaul V, Mamula P, Pedrosa MC, Rodriguez SA, ASGE Technology Committee, (2010). Enteral nutrition access devices. Gastrointest Endosc.

[7] Date RS, Clements WD, Gilliland R (2004). Feeding jejunostomy: is there enough evidence to justify its routine use?. Dig Surg.

[8] Srinathan SK, Hamin T, Walter S, Tan AL, Unruh HW, Guyatt G (2013). Jejunostomy tube feeding in patients undergoing esophagectomy. Can J Surg.

[9] Gupta V (2009). Benefits versus risks: a prospective audit. Feeding jejunostomy during esophagectomy. World J Surg.

[10] Alivizatos V, Gavala V, Alexopoulos P, Apostolopoulos A, Bajrucevic S (2012). Feeding tube-related complications and problems in patients receiving long-term home enteral nutrition. Indian J Palliat Care.

[11] Bose AC, Shankar RR, Kate V, Ananthakrishnan N (2005). Spontaneous antegrade enteral migration of feeding jejunostomy tube. Indian J Gastroenterol.

[12] Prahlow JA, Barnard JJ (1998). Jejunostomy tube failure: malnutrition caused by intraluminal antegrade jejunostomy tube migration. Arch Phys Med Rehabil.

[13] Polychronidis A, Karayiannakis AJ, Perente S, Botaitis S, Simopoulos C (2003). Enteral migration of a Pezzer tube after a feeding jejunostomy: report of a case. Surg Today.

[14] Ozben V, Karata A, Atasoy D, Sim ek A, Sarigul R, Tortum OB (2011). A rare complication of jejunostomy tube: enteral migration. Turk J Gastroenterol.

